# A qualitative study of patients' views on quality of primary care consultations in Hong Kong and comparison with the UK CARE Measure

**DOI:** 10.1186/1471-2296-10-10

**Published:** 2009-01-27

**Authors:** Colman SC Fung, Stewart W Mercer

**Affiliations:** 1 Department of Community and Family Medicine and School of Public Health, Chinese University of Hong Kong, Hong Kong, PR China; 2 General Practice and Primary Care, Division of Community-based Sciences, Faculty of Medicine, University of Glasgow, 1 Horselethill Road, Glasgow, G12 9LX, UK

## Abstract

**Background:**

Patients' priorities and views on quality care are well-documented in Western countries but there is a dearth of research in this area in the East. The aim of the present study was to explore Chinese patients' views on quality of primary care consultations in Hong Kong and to compare these with the items in the CARE measure (a process measure of consultation quality widely used in the UK) in order to assess the potential utility of the CARE measure in a Chinese population.

**Methods:**

Individual semi-structured interviews were conducted on 21 adult patients from 3 different primary care clinics (a public primary healthcare clinic, a University health centre, and a private family physician's clinic). Topics discussed included expectations, experiences, and views about quality of medical consultations. Interviews were typed verbatim, and a thematic approach was taken to identify key issues. These identified issues were then compared with the ten CARE measure items, using a CARE framework: Connecting (Care Measure items 1–3), Assessing (item 4), Responding (items 5,6), and Empowering (items 7–10).

**Results:**

Patients judged doctors in terms of both the process of the consultation and the perceived outcomes. Themes identified that related to the interpersonal process of the consultation fitted well under the CARE framework; Connecting and communicating (18/21 patients), Assessing holistically (10/21 patients), Responding (18/21 patients) and Empowering (19/21 patients). Patients from the public clinic, who were generally of lower socio-economic status, were least likely to expect holistic care or empowerment. Two-thirds of patients also judged doctors on whether they performed an adequate physical examination, and three-quarters on the later outcomes of consultation (in terms of relief or cure and/or side-effects of prescribed drugs).

**Conclusion:**

These findings suggest that Chinese patients in Hong Kong value engaged, empathic primary care doctors and judge the quality of consultations largely on these human skills and the attitudes and values that underpin them, as well as on the perceived outcomes of treatment. The match between themes relating to consultation process and the CARE Measure items suggests utility of this measure in this population, but further quantitative validation is required.

## Background

The consultation between doctor and patient is the core of clinical medicine and has been particularly emphasized in general practice and primary care [1-4]. Quality can be conceptualized as a combination of access to care and effectiveness of care, with effectiveness depending on both technical and interpersonal aspects [5]. Research on interpersonal effectiveness suggests that empathic, patient-centred consultations improve patient satisfaction [6,7] and enablement [8], and may improve health outcomes [9-11].

Although patient-centred care is becoming widely advocated, there is no single, globally accepted definition. Research on patient-centred care has predominantly been carried out in the West, especially the UK and North America [12,13]. Recently however, there has been a growing interest in people and patient-centred care in Eastern countries [14-17]. Additionally there is now a renewed focus on primary care globally, including countries such as China and Japan [18]. In seeking to develop effective primary care services in such countries, it is important that patients' views on what constitutes 'good' consultations are collected and fed into policy and health services developments in primary care. Hong Kong is one such region which is currently embarked on health care reforms with a strong emphasis on strengthening primary care services [19].

In the present qualitative study we have explored patients' views on what constitutes quality at consultation in different primary care settings in Hong Kong. We have then compared these views with the ten-item CARE measure, which is a widely used measure of consultation quality in the UK, developed by one of the authors [20-22]. The UK CARE Measure captures key aspects of the process of the clinical encounter, rather than outcome, and was developed from the views of patients of differing socio-economic status, as well as having a theoretical and empirical base. In a study of over 3,000 patients over 95% of patients felt the items were applicable to their consultation in primary care [22]. The CARE measure is currently accredited for appraisal of General Practitioners (GPs) and is a compulsory component of work place -based assessment in the training of all GPs in the UK.

## Methods

The study involved in-depth, semi-structured interviews with 21 Chinese patients recruited from 3 different types of primary care clinics in Hong Kong: (1) a Family Medicine Integrated Clinic (FMIC), part of the public healthcare system run by the Hospital Authority, (2) the University health centre (UHC) of the Chinese University of Hong Kong (CUHK), and (3) a private family medicine (FM) clinic run by a Family Physician specialist. The three settings were chosen in order to sample patients with a range of ages, conditions, and socio-economic status. The patients at FMIC mainly attend for chronic disease management such as hypertension or diabetes mellitus. Nine patients (5 males and 4 females), with age group ranged from 41–45 to 81–85, were recruited. UHC was selected because it was the health centre of CUHK and any student or staff and their family members can have access to it and many patients consult for acute problems. Six patients (1 males and 5 females), with age group ranged from <20 to 56–60, were recruited. The private FM clinic was selected because it was run by a family physician (a Fellow of the Hong Kong College of Family Physicians) and patients with either acute and/or chronic diseases commonly attend the clinic. Six patients (3 males and 3 females), with age group ranged from 26–30 to 76–80, were recruited. Ethics approval was obtained before the start of the study from the Survey and Behavioural Research Ethics Committee of the Chinese University of Hong Kong. The doctor-in-charge of each clinic consented to the study after receiving full details, and specific nursing staff at each clinic were assigned to facilitate the recruitment of patients in order to reduce the disturbance to the smooth running of the clinic. The nursing staff approached attending patients to see if they were interested in participating in an interview. After verbal agreedment, the first author approached the patient, explain the study in detail and obtained written consent. Keeping strict confidentiality and anonymity was emphasized to every interviewee. Although we were not able to purposively select the interviewees, recruiting from the 3 separate clinics helped to ensure that a maximum variation sample was obtained. Table 1 shows the characteristics of the 21 patients recruited.

**Table 1:** Characteristics of the participating patients

Patient Code	Gender	Age Group	Marital Status	Education Level	Occupation	Monthly Household income (HKD)	Reason/disease for consultation	Clinic Attended
A001	Female	71–75	Married	Tertiary	Retired	30001–40000	Chronic disease	FMIC
A002	Female	76–80	Widowed	Nil	Retired	20001–30000	Chronic disease	FMIC
A003	Female	71–75	Widowed	Nil	Retired	<5000	Chronic disease	FMIC
A004	Male	36–40	Single	Lower secondary	Non-technical manual worker	5001–10000	Chronic disease	FMIC
A005	Male	71–75	Married	Upper secondary	Retired	10001–20000	Chronic disease	FMIC
A006	Male	81–85	Widowed	Primary	Retired	<5000	Chronic + acute diseases	FMIC
A007	Female	66–70	Married	Primary	retired	<5000	Chronic + acute diseases	FMIC
A008	Male	76–80	Married	Primary	Retired	CSSA	Chronic disease	FMIC
A009	Male	41–45	Married	Lower secondary	Driver	10001–20000	Chronic disease	FMIC
A010	Female	56–60	Married	Upper secondary	Clerk	10001–20000	Acute disease	UHC
A011	Female	46–50	Married	Tertiary	Housewife	>60001	Acute disease	UHC
A012	Female	21–25	Single	Tertiary	Student	10001–20000	Administrative reason	UHC
A013	Female	46–50	Married	Tertiary	Clerk	>60001	Acute disease	UHC
A014	Female	<20	Single	Upper secondary	Student	10001–20000	Acute disease	UHC
A015	Male	21–25	Single	Tertiary	Student	30001–40000	Acute disease	UHC
A016	Male	71–75	Married	Nil	Retired	10001–20000	Chronic disease	Private
A017	Female	61–65	Married	Primary	Housewife	5001–10000	Chronic disease	Private
A018	Female	76–80	Widowed	Nil	Housewife	5001–10000	Chronic disease	Private
A019	Female	76–80	Widowed	Primary	Retired	5001–10000	Chronic disease	Private
A020	Male	26–30	Married	Tertiary	Officer	30001–40000	Acute disease	Private
A021	Male	31–35	Married	Tertiary	Professional	10001–20000	Acute disease	Private

FMIC = Family Medicine Integrated Clinic, Price of WalesHospital

UHC = University health centre, the Chinese University of Hong Kong

Private = private clinic of the Family Medicine Specialist

Patients were encouraged to talk freely and openly on their views on the quality of the primary care consultation in Hong Kong. Open-ended prompting questions included asking about their expectations, their experience of good or bad consultations, and their definitions of good or bad doctors/consultations. All the 21 interviews were conducted by the first author (CF), and a research assistant also helped to conduct the interviews on the six patients recruited from the private FM clinic. The interviews were conducted between May and August 2007 and averaged around 30 minutes (ranging from 15 minutes to 60 minutes).

The interviews were audio-taped and typed verbatim by a research assistant, and 4 student helpers (year 4 medical students of the Chinese University of Hong Kong). All transcripts were translated into English (all transcribers were native Cantonese speakers, fluently bilingual in written and spoken English). Accuracy of translation was checked by CF who read all transcripts in both English and Cantonese. Translation into English was necessary to enable SWM to assist in the analysis of the data.

A thematic approach was taken to identify key issues [23]. involving systematic identification, charting and sorting of the data. Analysis was iterative, with broad themes identified initially, then further broken down into sub-themes [24]. The constant comparative method was used throughout [25] by initially comparing data sets between individual transcripts, and later comparing data with emergent hypotheses. Regular meetings between the two authors over the duration of the project allowed categorisation and classification, and the development of typologies and explanatory accounts to be pursued.

**Figure 1:**
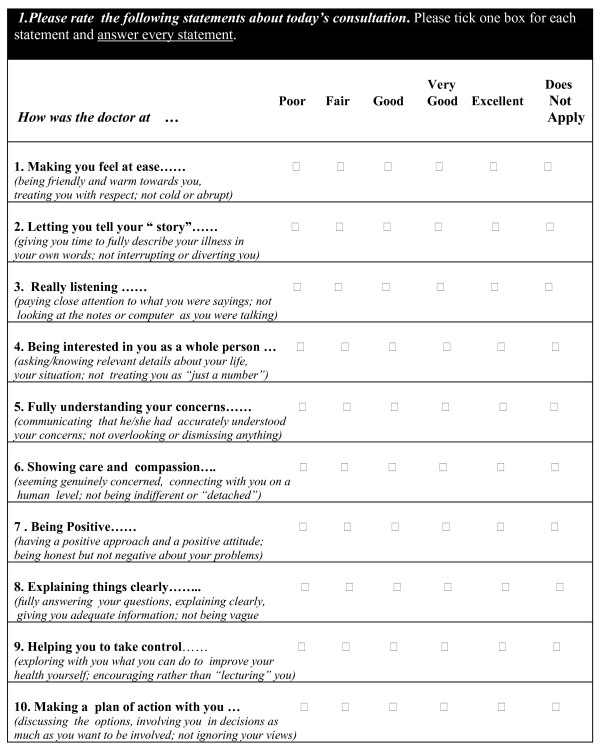
**The CARE Measure**.

Preliminary coding of the raw data was undertaken independently by CF and SWM and agreement reached on the initial main codes. Initially 16 categories were identified, which were then rearranged/re-coded under three major themes: patients' definitions of good doctors and consultations, bad doctors and consultations, and expectations/outcomes of the consultations. At this stage some 109 codes were generated in total. At the end of this process, the 109 codes were compared with the ten main items within the UK CARE measure (Figure 1). We then used the CARE Measure items as a framework for categorizing the codes identified regarding the consultation process, and assessed how many of the codes could or could not be categorized within one or more of the CARE Measure items. Categories not fitting within the CARE Measure framework were also identified. Both CF and SWM carried out this comparison with the CARE measure independently, and then compared notes; mainly there was good agreement, and areas of differences in if and where codes fitted within the framework were discussed until consensus was reached.

We found that all of the codes relating to the interpersonal aspects of the consultation (76 out of 109) fitted within one or more of the ten-item CARE Measure framework; Additional themes which did not fit (33 out of 109) related to physical examination, the context of care (access and time) and outcomes of care. Financial issues were also an important contextual issue, but will be reported in a separate paper. Rather than presenting our findings on interpersonal aspects of consultations under separate headings for all ten the CARE measure items, we have condensed these into 4 CARE Framework headings in order to present the findings in a more succinct manner;

1. Connecting and communicating (CARE items 1,2,3)

2. Assessing holistically (CARE Item 4)

3. Responding with understanding and compassion (CARE Items 5,6)

4. Empowering (CARE Items 7–10)

## Results

### Interpersonal care: The CARE Framework

#### Connecting and Communicating (CARE measure items 1,2,3)

A comfortable atmosphere within a consultation facilitated the patients' ability to relax and talk openly with the doctor. Patients felt that a polite manner, a warm welcoming smile, and words or gestures from the doctor at the onset of the encounter were means of putting them at ease.

"As I do not know much about medical knowledge, I can't tell what good element should be covered in a consultation. But at least sincerity. Sometimes, the doctor posed a not friendly facial expression, not even me, other patients, will also think that it is not good..." (A female patient aged 71–75, from the public primary healthcare clinic) [A001]

"I think it is better if the doctor smiles to patient. This shows care to patient, patient will feel better. You can't see some doctor smiling when you enter consultation room. It is not very good when the patient is feeling discomfort." (A female patient aged 56–60, from the UHC, CUHK) [A010]

Patients valued doctors who allowed or encouraged them to 'tell their story' and actively listened in the consultation, as it enabled them to describe their symptoms and problems in detail, and the effects these were having on their life. Some patients described such encounters as like 'chatting to a friend' and such a relationship enhanced disclosure of issues that were of importance to the patient.

"For me, I prefer those doctors who are more willing to talk to you and more willing to listen to you. Rather than those who just talk about, 'ok, this is the prescription, just take this medicine, you go back and take the medicine. If you are ok, then that's good; otherwise, you have to come back'..." (A female patient aged 46–50, from the UHC, CUHK) [A011]

Several patients equated "no eye contact" with "not listening" and disliked doctors who did not look at them but kept on writing or looking at the computer screen during the consultation.

"It's not good if the customer [patient] keeps telling, but the doctor just merely ask you what discomforts you have, then listens to the heartbeat and just says 'okay' and write down the things.. (A male patient aged 26–30, from the private clinic) [A020]

"...they usually face the computer and click this and click that, and then said, 'there's no problem, po-po [a form of address to the elderly female in Cantonese]. The blood pressure is normal. You can go to take the prescription now.' That's it. So I just left." (A female patient aged 71–75, from the public primary healthcare clinic) [A003]

18 out of the 21 patients interviewed had codes which fitted under this overall theme of 'connecting' (CARE item 1 = 13/21; CARE item 2 = 12/21; CARE item 3 = 8/21). The 3 who did not mention this overall theme were attending the private clinic and had above average household incomes.

#### Assessing holistically (CARE measure item 4)

'Whole person' care was seldom explicitly mentioned by patients as a key aspect of high quality interpersonal care, but many of their comments were integral to a holistic, bio-psycho-social approach. A few patients actually stated that they wanted doctors to spend more time understanding the possible psychological and/or social reasons behind their symptoms. For many patients, rather than 'volunteering' such issues, they felt a 'good' doctor would ask probing questions that would help uncover or 'dig-out' the underlying issues. This expectation that a whole-person approach should be 'doctor-led' reflected a widely held general view that a more assertive approach on the part of the patient would be impolite or rude.

"Of course it [asking questions] is important. We [the patient] only know what we should tell him/her [the doctor] if he/she asks us more questions. If he/she doesn't ask us, we don't know what we should say, right?" (A female patient aged 66–70, from the public primary healthcare clinic) [A007]

"They may explore the reason behind your stomachache, say, why you were suffering from stomachache last month but then 2 weeks later, you are suffering again? Is it due to something in daily life or something we eat, like sushi, milk?" (A female patient aged 46–50, from the UHC, CUHK) [A011]

"I think the most important thing for a good doctor is that he is willing to spend time to understand the patients' situation. It's not good if it is just like a routine in seeing all patients who go inside the consultation room." (A male patient aged 26–30, from the private clinic) [A020]

10 out of the 21 patients had codes which fitted under this theme. None of the patients attending the public clinic (FMIC) mentioned this theme, and only 1 male did.

#### Responding with understanding and compassion (CARE measure items 5,6)

Many patients considered understanding concerns, being caring, kind, compassionate, having love, were basic attributes of a good doctor. When such behaviours were expressed by doctors, patients deeply appreciated them. Such doctors were regarded as more trustworthy, and patients felt more able to talk openly about their concerns and fears in such encounters. Only a few patients mentioned the education and training of the doctors as an important factor in deciding whether a doctor was good or not. Instead, the perception of caring from the doctor during the consultation was a key criterion that they used to differentiate whether a doctor was 'good or bad'.

"A good doctor should have love. It's the hardest time for one when one is sick. Poor is not the toughest, but being sick is the toughest." (A female patient aged 76–80, from the private clinic) [A019]

"Also doctors have to care about the patients. When patients feel that the doctor cares about you, wants to know more about your condition, patients will talk more spontaneously." (A female patient aged 21–25, from the UHC, CUHK) [A012]

"This doctor has a 'real heart' [enthusiasm] to treat patients. It's different from other doctors. He will try all his best to treat you if you can be treated. It's different from other doctors who are aiming at making more money, and they will still 'treat' you no matter you are treatable or not." (A male patient aged 71–75, from the private clinic) [A016]

18 out of the 21 patients had codes under this overall theme (CARE item 5 = 13/21, CARE item 6 = 12/21). The 3 who did not showed no particular pattern.

#### Empowering (CARE measure items 7–10)

Many patients felt a 'good doctor' was one with a positive approach, which helped to give them hope. Being positive was also often associated with the doctor giving direct advice, a clear diagnosis, treatment, and the likely time-course of outcome from treatment. This theme was closely related to the theme of explaining things clearly, which almost most patients interviewed raised as a key feature of a good doctor or a good consultation. The importance of explanations to patients was not related to whether they attended the public or the private healthcare sectors.

"When I come for follow-ups, the doctors will tell me in details, tell me about my condition, and let me know it. It's good if they do it in this way. Something like giving advice to me on avoiding certain kinds of food, or what kind of food I should not eat, what kind of food I can eat." (A male patient aged 41–45, from the public primary healthcare clinic) [A009]

"...Besides, being a doctor is like being a teacher...they should encourage student or patient what they should do, because they will accept this easier." (A female patient aged 21–25, from the UHC, CUHK) [A012]

Giving advice or educating patients about what they could do to help themselves was another area that was highly valued by patients. Domains of advice included how to improve one's health, advice on preventive care, self-help measures, dietary and lifestyle advice. In general patients gave high importance to the doctor's role in 'education' in addition to making a diagnosis and prescribing drugs.

"If so, patients can learn what they can do to self-help themselves. You know, with the help of the doctor, you can help the patient to cope with his/her own symptoms so that he/she doesn't have to see doctor so often.." (A female patient aged 46–50, from the UHC, CUHK) [A011]

"For example, he (the doctor) can explain and describe your disease in an easily understandable way. He will also teach you what kinds of exercises that you should do, and will show you on how to do those stretching exercises on shoulder... He explains your disease well and in a detailed manner. He will also give me some pamphlets to see so that I can know more about my disease as well." (A male patient aged 31–35, from the private clinic) [A021]

Only two out of twenty-one patients voiced a clear desire for shared decision making between doctor and patient (one was from the University Health Centre and one was from the private clinic).

"The point is doctors have given me their professional advice and it's up to me to decide. If I need antibiotics, the doctors can give me, otherwise, observation is recommended. It's fine. We can still do something at home rather than always using some strong western medication as there must be some side effects...the doctors may not have time to explain all those to you." (A female patient aged 46–50, from the UHC, CUHK) [A011]

"He (the doctor) will not force me. Even if he tells me that there is a (special) situation, he will not say something like ' you have to do it.'..." (A female patient aged 61–65, from the private clinic) [A017]

However, in other cases, it seemed that the patients' views on decision were not considered by doctors, even though this may have been desirable by the patients.

"Sometimes there are some ointments that I think they are effective on me, but the doctors usually refuse to prescribe them to me." (A male patient aged 36–40, from the public primary healthcare clinic) [A004]

Another patient from the public healthcare sector explained that she did not expect the doctor to listen to the patient's opinion in decision making but she valued the regular follow-up.

"You can come here regularly for body check, do some blood tests, so you will know if there's any problem with you. They won't do this to you if you go to see the doctors elsewhere, they won't do such checking for you to see if you have any problem. No, no such things. But it is here, it's better to have such checking in the long term....If the doctor asked me, 'is it okay to add some more to the drug dosage?' I usually say, 'It doesn't matter for me, if you think it's good for me.' When I go to see doctor, I usually listen to what the doctor says. There's no reason for the doctor to listen to my sayings, right? If he wants to add some more, then I just say 'okay'. (A female patient aged 76–80, from the public primary healthcare clinic) [A002]

19 out of 21 patients had codes under this overall theme (CARE item 7 = 11/21, CARE item 8 = 18/21, CARE item 9 = 10/21, CARE item 10 = 5/21). For items 7, 9, 10, patients attending the public clinic (FMIC) were in the main those who did not mention these themes.

### Physical examination

Apart from those domains within a consultation that patients had mentioned above, it was interesting to note that almost one-third of patients from each type of clinic pointed out that they valued the act of physical examination. They perceived physical examination as an attribute of a caring and thorough doctor, and an important part of diagnosis, and hence getting a 'full-picture' of the problem.

"For example I have abdominal pain, he [the doctor] will think about my family history, and consider whether I am having that disease or not, then do a detail examination for me. Some doctors... abdominal pain, they will only give you some pain killers. You can feel that which doctor cares about you more. (A female patient aged 56–60, from the UHC, CUHK) [A010]

"The private doctors are more considerate. They will do some examinations for you, but not here, they will just take a look and no examination. The private doctors will examine you, measure the blood pressure......but not here. They will just say, 'put some ointments and get some medicines back home.' and tell me when I should return for follow-up." (A female patient aged 66–70, from the public primary healthcare clinic) [A007]

"Some doctors are very meticulous. They examine the nose and throat carefully and answer my query, so I feel satisfied and think that they are good doctors. I remember an occasion in which a doctor listened to my son's chest and examined his ear and throat, and told me about his findings. I got a full picture of my son's illness. I found this doctor very good, very meticulous. Some doctors just glance at my son's face and take less than a minute examining my child. I find them very sloppy." (A female patient aged 46–50, from the UHC, CUHK) [A013]

### Context of care

#### Access

Approximately one-third of patients (from each type of clinic) complained about having to wait too long before their consultations.

"...it took me two hours waiting until I saw the doctor. That's really bad. I got herpes zoster that time. It was really painful..." (A female patient aged 76–80, from the public primary healthcare clinic) [A002]

"If you see public doctors, it will be impossible that you can see them whenever you want. You won't be able to see [public doctors] if you miss your turn." (A female patient aged 76–80, from the private clinic) [A018]

#### Time spent on consultations

Some patients specifically mentioned the issue of consultation length, and resented the fact that some doctors spent very little time with them, quickly writing a prescription with no explanation. Feeling rushed in consultations and thus an inadequate consultation length, was implicit in many of the above accounts relating to interpersonal quality of care.

"I think if the consultation time is longer, they (the doctors) can have more understanding on the patients' symptoms. I think there are lots of reasons behind a symptom. Say, you may think you are suffering from stomachache; in fact, it may not be that simple, it may be due to the stress in daily life. So, I don't know, sometimes when the doctors are busy, they may just prescribe pain killers, 'ok, that's it, byebye.'..." (A female patient aged 46–50, from the UHC, CUHK) [A011]

#### Continuity

Continuity of care was not overtly discussed in the interviews, but lack of relational continuity was apparent in the accounts of the patients attending the public healthcare system, where it is unusual for patients to be seen by the same doctors. Similarly, 'doctor-shopping' in the private sector is a common phenomenon in Hong Kong. However, when patients felt they had a good 'match' with a doctor, they were keen to continue seeing that doctor if at all possible.

#### Outcomes

Two-thirds of the patients linked a good consultation with the doctor making a correct diagnosis leading to a rapid "cure" of his/her disease or illness. Thus judgments about quality of consultation and doctor were retrospective based on outcomes; if the patient recovered rapidly then they perceived the consultation, the doctor, and the treatment, as effective and thus of high quality.

"The doctor is very kind, and he can often make a correct diagnosis." (A female patient aged 76–80, from the private clinic) [A019]

"If such questions help doctors in making the diagnosis, I don't mind to answer them. But if the doctors think that they know enough about my condition without the need to ask such questions, then that's ok. To me, the most concerning point is that the doctor can solve the problem and treat my disease." (A male patient aged 21–25, from the UHC, CUHK) [A015]

Many patients expressed dissatisfaction if their diseases or illness symptoms did not disappear quickly. Many of them viewed the drugs given by the doctors as an important factor as to whether they would get better or not.

"If his medicine was efficient, I would go back to see him (the doctor), otherwise I won't go back." (A female patient aged 71–75, from the public primary healthcare clinic) [A003]

"The medicine private doctors gave me ... I can recover faster after I have taken the medicine and have the injection." (A male patient aged 36–40, from the public primary healthcare clinic) [A004]

"He (the doctor) will not lengthen your treatment, and you are able to get well in a short time...he is really a good doctor... he can treat my diseases, then that means good." (A male patient aged 71–75, from the private clinic) [A016]

Some even judged the 'relationship' on the 'outcome' of the consultation rather than on the process of the consultation.

"If he can cure me, then that's a good relationship. If he cannot cure me, then the relationship will not be able to build up and continue." (A male patient aged 26–30, from the private clinic) [A020]

## Discussion

In the present study we assessed patients' views on the quality of the primary care consultation in Hong Kong by means of qualitative interviews of 21 patients attending three different types of primary care clinics (public clinic, University clinic, and private family medicine clinic) and tested whether these views are similar or different from patients views in the UK, by comparing the themes identified from the present study with the themes that comprise the UK CARE Measure. Patients judged doctors in terms of both the process of the consultation and the perceived outcomes. Themes identified that related to the interpersonal process of the consultation fitted well under the four theme CARE framework that we devised to incorporate the ten CARE Measure items; connecting and communicating (CARE items 1–3), assessing holistically (CARE item 4), responding with understanding and compassion (CARE items 5,6) and empowering (CARE items 7–10).

As far as we are aware, this is the first qualitative study of patients' views on consultation quality among Chinese patients attending a variety of different primary care providers in Hong Kong. Although international differences in patient and physician perceptions of "high quality" healthcare have been reported [26] and despite the many cultural differences between the East and the West, the core aspects of consultation quality in primary care as expressed by Hong Kong patients in the present study appear to be broadly similar to studies in Caucasian subjects in the West [27,28].

However, there did appear to be some differences in the way Chinese patients in the present study 'accessed' high quality consultations compared with studies in the West. Directly asking for information and advice was uncommon; rather patients waited for such advice and information to be 'offered' by the doctor. Similarly, with respect to a holistic approach to care, patients wanted doctors to 'dig-out' their problems, rather than assert them themselves. Similarly, patients had a low expectation, and apparent desire for, shared decision making in the consultation. These differences, which at face value suggest that Chinese patients are somewhat passive in medical consultations may relate to cultural factors and/or to a significant hierarchy and power differential between patients and doctors in Hong Kong. On the other hand, many of the patients in the present study were elderly and of lower socio-economic status. In the UK, although shared-decision making is a key policy and educational objective in medicine, several studies have reported low expectation of/desire for shared-decision making in older patients and patients of lower socio-economic status [29,30]. Similarly, another European study reported that elderly patients define involvement in care more in terms of the caring relationship and information receiving rather than on active participation in decision making [31].

Patients from the public clinic, who were generally of lower socio-economic status, were least likely to expect holistic care or empowerment in the present study. Further work is required to explore this, but it may relate to a more biomedical approach in these clinics, which deal mainly with chronic diseases. In the UK patients of lower socioeconomic status gain less enablement from consultations [32] especially if the clinical issues are complex [33] and in the USA greater dissatisfaction with health care amongst low-income patients has been reported with such patients feeling not listened to and 'brushed off by physicians [34].

In addition to these interpersonal aspects of care, these Hong Kong Chinese patients placed a high regard of receiving a physical examination. One reason for this may be that in Traditional Chinese Medicine (which is very commonly used by the Hong Kong population), physical examination such as looking at the tongue, face, and palpating the peripheral pulses is an integral part of a consultation and diagnosis. Given that TCM is still commonly used by people in Hong Kong.

Contextual issues of access to care, continuity, and consultation length interacted with opinions of 'good consultations'. Such judgements perhaps need to be seen within the general context of primary care in Hong Kong and the 'doctor-shopping behaviour' that is common place, at least in the private sector [35]. The fact that patients judged 'good consultations' not just on interpersonal aspects but also retrospectively according to outcome (and hence perceived effectiveness of treatment) has also been reported in the UK [29] but may be of greater importance to patients in Hong Kong given that most primary care is private requiring out-of-pocket payment.

The present study also had limitations. Because of time constraints we limited the number of interviews to 21 and thus we cannot be sure that data saturation was reached regarding all themes. The three clinics selected to recruit patients for interviews were not randomly selected and were geographically situated in the New Territories of Hong Kong, which is generally a less affluent area than Hong Kong Island. Nonetheless, our sampling frame did include a range of patients of differing ages, gender, socio-economic status, and disease states. The aim of this study (and indeed of all qualitative studies) was not to generate findings that can be said to be representative of the general population, but to identify themes relating to consultation quality that can be tested in larger, quantitative studies. In this respect, we feel the present study has been successful, and the fact that the key interpersonal aspects of the consultations identified matched the items contained in the CARE Measure paves the way for further work on translation of the CARE measure into Chinese and validation studies. If the Chinese-CARE Measure proves to be a feasible, acceptable, and robust tool it may have wide-spread utility in the formative and/or summative assessment of medical students and primary care doctors in Hong Kong and mainland China, as well as in future research on consultation quality.

## Conclusion

In conclusion, the results of the present qualitative study on patients' views on consultation quality in primary care suggest that Chinese patients in Hong Kong value engaged, empathic primary care doctors and judge the quality of consultations largely on these human skills and the attitudes and values that underpin them, as well as on the perceived outcomes of treatment.

## Competing interests

The authors declare that they have no competing interests.

## Authors' contributions

SM will act as the guarantor for the study. SM and CF conceived and designed the study. CF collected data and carried out an initial analysis and interpretation of the data. SM helped in the secondary analysis and interpretation of the data. CF accomplished the first draft. SM revised several versions of the manuscript with CF, and also gave critical intellectual input into this process. All authors read and approved the final manuscript.

## Pre-publication history

The pre-publication history for this paper can be accessed here:


